# Electrochemical Immunosensor for the Early Detection of Rheumatoid Arthritis Biomarker: Anti-Cyclic Citrullinated Peptide Antibody in Human Serum Based on Avidin-Biotin System

**DOI:** 10.3390/s21010124

**Published:** 2020-12-28

**Authors:** Somasekhar R. Chinnadayyala, Sungbo Cho

**Affiliations:** 1 Department of Electronic Engineering, Gachon University, 1342 Seongnamdaero, Seongnam-si, Gyeonggi-do 13120, Korea; ssreddy@gachon.ac.kr; 2 Department of Health Science and Technology, GAIHST, Gachon University, Incheon 21999, Korea

**Keywords:** anti-cyclic citrullinated peptide antibodies, interdigitated chain shaped microelectrode array, rheumatoid arthritis, immunosensor, electrochemical impedance spectroscopy

## Abstract

Rheumatoid arthritis (RA) is a chronic autoimmune disease that produces a progressive inflammatory response that leads to severe pain, swelling, and stiffness in the joints of hands and feet, followed by irreversible damage of the joints. The authors developed a miniaturized, label-free electrochemical impedimetric immunosensor for the sensitive and direct detection of arthritis Anti-CCP-ab biomarker. An interdigitated-chain-shaped microelectrode array (ICE) was fabricated by taking the advantage of microelectromechanical systems. The fabricated ICE was modified with a self-assembled monolayer (SAM) of Mercaptohexanoic acid (MHA) for immobilization of the synthetic peptide bio-receptor (B-CCP). The B-CCP was attached onto the surface of SAM modified ICE through a strong avidin-biotin bio-recognition system. The modified ICE surface with the SAM and bio-molecules (Avidin, B-CCP, Anti-CCP-ab and BSA) was morphologically and electrochemically characterized. The change in the sensor signal upon analyte binding on the electrode surface was probed through the electrochemical impedance spectroscopy (EIS) property of charge-transfer resistance (R_ct_) of the modified electrodes. EIS measurements were target specific and the sensor response was linearly increased with step wise increase in target analyte (Anti-CCP-ab) concentrations. The developed sensor showed a linear range for the addition of Anti-CCP-ab between 1 IU mL^−1^ → 800 IU mL^−1^ in phosphate buffered saline (PBS) and Human serum (HS), respectively. The sensor showed a limit of detection of 0.60 IU mL^−1^ and 0.82 IU mL^−1^ in the PBS and HS, respectively. The develop bio-electrode showed a good reproducibility (relative standard deviation (RSD), 1.52%), selectivity and stability (1.5% lost at the end of 20th day) with an acceptable recovery rate (98.0% → 101.18%) and % RSD’s for the detection of Anti-CCP-ab in spiked HS samples.

## 1. Introduction

Nearly 1% of the world’s population is affected by RA, a chronic autoimmune and degenerative disease [1,2,3]. The exact cause for the generation of autoimmune antibodies (AAb) is still unknown, but it is believed that the triggering mechanism caused by genetic (HLA-DR1 and HLA-DR4) and environmental (smoking and pathogens) factors producing autoimmune antibodies attack their own cells [4,5]. In brief, the citrullination of peptides in Type-II collagen and vimentin produces Citrulline in the place of arginine [6]. Meanwhile, the production of IgM Rheumatoid factor (RF) and Anti-cyclic citrullinated peptide/protein antibodies (Anti-CCP-ab) produces immune complexes with specific antigens that accumulate at the inflamed joints. Early detection of RA indeed plays a vital role in preventing irreversible joint erosion. Almost 70% of the people with a positive Anti-CCP-ab in their serum have a 5-year risk of developing RA [7]. The impact of anti-rheumatic therapy can be assessed by evaluating the level of Anti-CCP-ab along with RF factor [8,9,10]. Furthermore, because of the early presence of Anti-CCP autoantibodies and their higher specificity, Anti-CCP-ab’s can serve as superior diagnostic markers in the early detection of RA. The normal level of Anti-CCP-ab is considered to be <20 IU/mL and the cut-off values of Anti-CCP-ab level have been set at 20 IU/mL for RA. For the detection of Anti-CCP-ab’s, citrullinated vimentin, citrullinated a-enolase, and citrullinated fibrinogen can be used as the discerning antigens for diagnosing RA. Cyclic citrullinated peptides (CCPs) are the most common selective antigens for recognizing Anti-CCP-ab’s [11]. Present day diagnostic techniques for Anti-CCP-ab detection include enzyme-linked immunosorbent assay (ELISA) [12,13], surface-enhanced Raman scattering [14,15], chemiluminescence analysis [16,17], fluorescence-based analysis [18], and electrochemical analysis. An increase in the titer concentration of Anti-CCP-ab’s can serve as a predictive tool for RA and provide therapeutic information about the effectiveness of the treatment.

The fabrication of electrochemical antibody detection biosensors, for the quantitative analysis and monitoring of disease biomarkers, is presently an emerging field of research activity. Recently, a few electrochemical biosensors for the sensitive detection of antibodies for SARS-CoV-2 spike protein [19], Zika virus specific antibodies [20], glutathione (GSH) monoclonal antibody [21], Human IgG [22] and anti-infiximab antibody [23] were reported. In spite of the substantial developments provided by the electrochemical biosensors, only a few bio-sensing devices are available for Anti-CCP-ab detection. Recently, Guerrero et al. developed an electrochemical sensor based on neutravidin-functionalized magnetic microbeads-modified screen-printed electrode (SPE) for aCCP detection in human serum, where a linear dynamic range of 10–1000 IU/mL with a limit of detection (LOD) of 2.5 IU/mL for aCCP was reported [24]. Villa et al. reported a biosensor specific for anti-CCP-ab based on multi-walled carbon nanotube–polystyrene electrochemical transducer. The sensing platform was covalently immobilized with a chimeric fibrin-filaggrin synthetic peptide as the bio-receptor through amide coupling between the synthetic peptide and terminal–COOH functional groups of MWCNTs. The developed biosensor was applied for the detection of Anti-CCP-ab in HS [25]. Thus, the accurate, selective and sensitive quantification of Anti-CCP-ab’s in HS with significant linear ranges, detection limits for rapid, reliable, and precise detection of the AAbs for early diagnosis of RA based on electrochemical transducer is of prime importance.

A well-ordered bio-sensing platforms has attracted much attention for applications to analytical devices. Self-assembled monolayers based on –COOH terminal alkanethiols offered the easiest ways to develop well-ordered, reproducible and oriented thin-films, that can pertain the activity of biomolecules [26,27,28]. SAM-based alkanethiols on gold substrates have been widely used than others because -SH molecules are well bound to gold surface [29]. Biomolecules can be attached on properly functionalized SAMs through covalent bond formation by reacting with functional groups exposed on side-chains of the biomolecules [30]. Usually, lysine side chains of protein (amine groups) would react with succinimide-esters of carboxylic acids of a SAM. Robust and non-covalent interactions such as streptavidin–biotin, neutravidin–biotin or avidin–biotin interaction systems have frequently been used to immobilize biomolecules to SAM surfaces [31,32,33]. The bond formation between avidin–biotin (K_d_ = ~10^−15^ M), is rapid, unaffected by pH, temperature, organic solvents and was used to attach biomolecules to electrode surface [34].

Recently, label-free electrochemical impedance spectroscopy (EIS)-based sensing based on interdigitated micro electrode arrays is gaining substantial interest for sensitive quantification of target bio-analytes [35,36,37]. EIS is a non-destructive and sensitive technique utilized for the characterization of modified electrodes and for the analysis of electrochemical systems and processes. In addition, an electrochemical biosensor based on EIS provides several benefits such as low power consumption, and ease of miniaturization. EIS biosensors provides signal out-put by using periodic small AC perturbations and responds to signal change caused by the binding of bio-analytes to the immobilized bio-recognition elements on the electrode surface [38]. Due to these advantages an impedance based biosensor finds a suitable position compared to the other electrochemical transduction mechanisms for developing miniaturized point-of-care-testing (POC) applications.

Furthermore, advancements in MEMS has introduced micro-/nanoelectrode’s and miniaturized sensing transducers in EIS based transducer platforms for selective, fast and sensitive detection of protein/peptide biomarkers [39,40]. The MEMS fabrication of an interdigitated chain shaped micro electrode array with a simple electronic circuitry for impedance immunosensing of Anti-CCP-ab provides mass production of electrode arrays with low cost and sample loadings. By considering the collective advantages of aforementioned techniques (SAM, MEMS and EIS), has made the present electrochemical immunosensing strategy competitive with other CCP detection immunosensing techniques.

**Figure 1:**
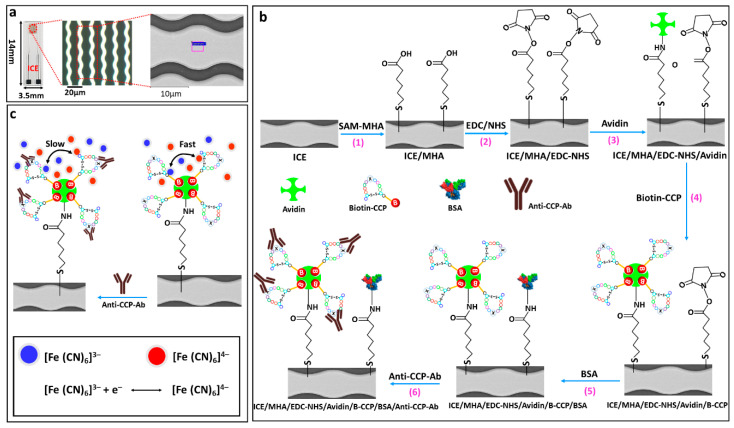
Optical and microscopic images of the fabricated ICE arrays showing a uniform width and spacing (**a**). The schematic illustration of the electrode surface modification with MHA-SAM, EDC-NHS coupling chemistry for avidin immobilization, avidin-biotin bio-recognition system for synthetic peptide (CCP) binding on electrode surface as bio-receptor, BSA binding on electrode surface to prevent non-specific binding of the bio-analyte of interest (Anti-CCP-ab) (**b**). Mechanism of EIS sensing of rheumathritis biomarker, Anti-CCP-ab in [Fe(CN)_6_^3−/4−^] electrolyte probed through the sensitive changes in electrode interfacial property (R_ct_) upon bio-analyte binding on the modified electrode surface (**c**).

In the present study, we developed an impedimetric immunosensor by taking the advantage of avidin–biotin interaction systems to attach the cyclic-citrullinated peptide (CCP) on the surface of SAM modified (6-mercaptohexanoic acid) interdigitated chain shaped microelectrode arrays (ICE). As a proof-of-concept, a synthetic peptide (biotin conjugated cyclic-citrullinated peptide; B-CCP) was selected as the bio-receptor to immobilize on the transducer surface for selective detection of the arthritis AAb (Anti-CCP-ab). The change in sensor response upon analyte binding on electrode surface is probed through the electrode interfacial property (charge transfer resistance; R_ct_) of EIS. The sensor was miniaturized (14 × 3.5 mm) and can be combined to fabricate a portable immunosensing device to detect Anti-CCP-ab for POC testing. The developed immunosensor (ICE/MHA/EDC-NHS/Avidin/Biotin-CCP/BSA) can selectively detected Anti-CCP-ab in HS within 10 min. Furthermore, the avidin-biotin bio-recognition system and SAM of MHA-modified ICE surface provided an excellent platform for the detection of rheumatoid biomarker by preserving the bio-activity of the immunogen (B-CCP). Based on the available studies and to best of our knowledge, this is the first report on miniaturized ICE configuration for rheumatoid marker detection in PBS and HS samples. The mechanism of the impedance sensing and schematic representation of the major electrode modification steps are presented in Figure 1.

## 2. Materials and Methods

### 2.1. Chemicals and Reagents

Biotin-labelled cyclic citrullinated peptide (Biotin-CCP) (4166 IU/mL) (Peptide sequence: Biotin-HQCHQEST-Cit-GRSRGRCGRSGS-COOH) with a disulfide bond between the Cyc3 and Cyc16 residues was procured from Peptron Inc. (Daejon, South Korea). Anti-cyclic citrullinated peptide antibody (Anti-CCP-ab) (4160 IU mL^−1^) was procured from Bioss Antibodies Inc. (Woburn, Massachusetts, USA.). 6-Mercaptohexanoic acid (MHA), Hemoglobin, Potassium ferrocyanide (K_4_[Fe(CN)_6_]·3H2O), potassium ferricyanide (K_3_[Fe(CN)_6_]), 1-ethyl-3-(3-dimethylaminopropyl)-carbodiimide (EDC), N-Hydroxysuccinimide (NHS), Avidin from egg white (≥10 Units/mg protein, ≥98%), Bovine serum albumin (BSA), and human serum were purchased from Sigma-Aldrich (USA). Anti-Interferon-γ-antibody (Anti-IFN-γ-ab), Anti-insulin-antibody (Anti-insulin-ab), IgM-Rheumatoid factor (IgM-RF), Anti-C-reactive-protein-antibody (Anti-CRP-ab), Human-Immunoglobulin-G-antibody (Human-IgG-ab) were procured from Abcam (Cambridge, UK). Phosphate buffered saline (PBS- 137 mM NaCl, 2.7 mM KCl, 4.3 mM Na_2_HPO_4_, and 1.4 mM KH_2_PO_4_; pH 7.4) and 1 × PBS containing 0.05% Tween 20 were procured from Tech and Innovation (Gangwon, South Korea). All solutions were prepared using deionized water (DI; 18.2 MΩ cm) from Purescience (Jungwon, South Korea). All other chemicals were of analytical reagent grade and are used without any further purification.

### 2.2. Instrumentation

X-ray photoelectron spectroscopy (XPS) elemental surface analysis was carried out using a PHI 5000 Versa Probe (Ulvac-PHI) spectrometer (Japan) with monochromator A1 Kα (1486.6 eV). Survey scan was first recorded and then region scans were measured for the S(2p) and C(1s) photoelectron binding energy regions. A 50 eV band pass energy, 1 eV step size and 200 μm × 200 μm X-ray spot size were used for measuring survey scan (range = 1200 to −5 eV). For C1s and S2p region scans a pass energy of 20 eV and 0.1 eV step size were used. All the region scans were fitted by a standard Gaussian curve fit with Shirley background subtraction [41]. Atomic force microscopy (AFM) measurements were obtained on an ambient air scanning probe microscope (XE–100 Park systems, South Korea). A typical non-contact mode was used to record the images using the XEP software. The scanning region was approximately 2 × 2 µm^2^, with a resolution of 0.05 nm and scan rate of 0.5 Hz. The surface morphology and composition of elements were investigated using scanning electron microscopy (Hitachi S-4700 (Japan) with an operating accelerated voltage of 15 kV and energy dispersive X-ray.

### 2.3. Electrochemical Measurements

Cyclic voltammetry (CV) was carried out in a three-electrode configuration (CompactStat potentiostat, Eindhoven, Netherlands) consisting of a working electrode (WE) (bare and modified ICEs), a counter electrode (CE) (Platinum coil), and a reference electrode (RE) (Ag/AgCl). The cyclic voltammograms (CVs) were carried out in 5 mM [Fe(CN)_6_]^3−^ in 1 × PBS (10 mM, Phosphate buffered saline) (pH 7.4), at a sweep rate of 50 mV s^−1^ within a potential range of −0.2 V to +0.5 V vs. Ag/AgCl external RE. The electrochemical impedance spectroscopy (EIS) measurements were carried out in 5 mM [Fe(CN)_6_]^3−^/^4−^ (1:1) in 1 × PBS, at room temperature (RT) (24 °C) using a two-electrode setup. The spectra were measured by selecting an alternating current perturbation voltage with a root mean square value of 0.05 V in a frequency range of 0.1 Hz–1 MHz and three points were collected for a decade of frequency. The measured impedance spectra were fitted by using a suitable equivalent circuit model consisting of solution resistance (R_s_), electrode interfacial capacitance (CPE) and charge transfer resistance (R_ct_). The fitting of the EIS spectra was carried out by ZView-a non-linear curve fitting software from Scribner Associates Inc. (Southern Pines, NC, USA).

### 2.4. Fabrication of the ICE Arrays

A gold (Au) based ICE array was fabricated on a glass slide substrate (14 × 3.5 × 0.5 mm^3^). The conductive Titanium and Au (thickness: 25 and 50 nm, respectively) layers were deposited by an electron beam evaporator. Subsequently, a pair of conductive pads and interdigitated chain shaped electrode fingers, consisting of 5 µm spacing and width for WE and CE/RE were developed by the lift-off process. For measuring electrochemical impedance spectra, a polystyrene cell culture plate (96-well) with 400 μL volume capacity was used as an electrolyte reservoir. A homemade adapter was fabricated to connect the WE and CE/RE conductive pads of ICE to the potentiostat.

### 2.5. Preparation of the Anti-CCP-ab Bioelectrode

The ICE electrode array was washed with successive solutions of ethanol (99.5%), and water for 2 min via ultra-sonication and purged under N_2_ gas to remove surface bound impurities. The SAM was immobilized on the electrode surface by incubating the electrodes in 50 mM MHA prepared in 99.5% ethanol for overnight (12 h) at room temperature. The MHA modified electrode was rinsed with pure ethanol followed by DI water to remove loosely bound thiol moieties. Additional rinsing was carried out to remove the H-bonded thiol moieties by sonicating the electrode in absolute ethanol for 2 min. After sonication, the electrodes were washed with DI water dried under low stream of N_2_ gas. The terminal –COOH groups of the MHA-SAM modified electrode was activated by EDC (75 mM)/NHS (5 mM) solution (30 min incubation at room temperature) for Biotin-CCP binding. The EDC-NHS activated electrodes were washed by 1 × PBS followed by DI water. 10 µL of 100 µg mL^−1^ of avidin was dropped onto the surface of MHA/EDC-NHS electrode and incubated for 1 h in the humid chamber to prevent drying of the electrode surface. The avidin was immobilized covalently by a coupling reaction between the –NH_2_ group of the avidin and the EDC-NHS activated MHA moieties on the electrode surface. After avidin immobilization the electrodes were washed with 1 × PBS to remove the loosely bound avidin. 10 µL of 50 µg mL^−1^ of Biotin-CCP was dropped on the electrode surface and incubated in humid chamber for 30 min. The Biotin-CCP added is immobilized on the electrode surface through avidin-biotin bio-recognition system. The electrodes (ICE/MHA/EDC-NHS/Avidin- Biotin-CCP) formed were washed with 1 × PBS to remove any unbound Biotin-CCP. Subsequently the electrodes were washed with 1 × PBS buffer containing 0.05% Tween-20 and 0.5% BSA for 2 min to prevent the non-specific binding of the target protein, thus forming ICE/MHA/EDC-NHS/Avidin/B-CCP/BSA bio-electrode.

## 3. Results

### 3.1. Characterization of SAM-MHA Functionalization on ICE

**Figure 2:**
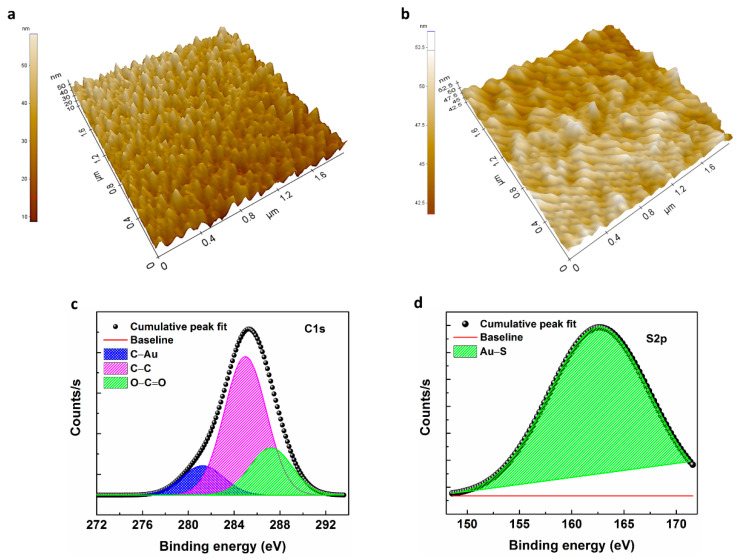
AFM topography images of the ICE electrode array before (**a**) and after (**b**) SAM functionalization. XPS spectra of the ICE electrode array modified with SAM-MHA: C1s (**c**) and S2p (**d**) region scans fitted using Gaussian cure fit with shrilly background subtraction.

The Energy dispersive x-ray spectroscopy analysis (EDX) were performed to confirm the atomic ratios and elemental composition of the modified electrodes (SEM/EDX), by carrying out measurements over the randomly selected areas. The EDX of the bare ICE surface shows almost Au (due to 50 nm Au coating) and minor amounts of Si, O and C (due to the underlying glass substrate) (Figure S1a). The elemental composition of the ICE/MHA shows major quantities of C, O (due to the MHA functionalization) and Au stemming from gold substrate, and minor quantities of S from MHA-SAM (Figure S1b). AFM topography images were recorded to study the surface morphology of the electrode functionalized with MHA-SAM. Figure 2a shows the polycrystalline surface of the bare gold ICE surface with an average roughness of 3.753 nm. The electrode modified with MHA-SAM was changed into a smooth topography and homogenous aligned structure with an average roughness of 1.18 nm when compared to the bare ICE (Figure 2b). The results demonstrate the successful modification of bare ICE electrode surface with MHA-SAM.

To analyse the SAM functionalization on the ICE surface, elemental dispersive X-ray and elemental mapping analysis was performed on the ICE/MHA electrodes. The survey spectrum for ICE/MHA electrodes show peaks corresponding to C, S and Au (Figure S2). The high-resolution region scans of C1s and S2p for ICE/MHA along with the respective peak fitting components are shown in Figure 2c,d. Binding energies (BEs) of carbon (C1s) are observed at 285.01 eV, 287.22 eV and 281.24 eV (Figure 2c). The main peak detected at 285.01 eV correlates to the adventitious carbon. The shoulder peak at 287.22 eV is ascribed to the O−C=O functional group. The peak at BEs 281.24 eV endorsed to Au-C interaction [42]. All the peaks were normalized by shifting of the C–C peak to 285 eV. The S2p deconvolution region scans show a distinct peak at 161.70 eV corresponding to the Au-S interaction in the ICE/SAM surface [43].

### 3.2. Optimization of the Immunosesnor

**Figure 3:**
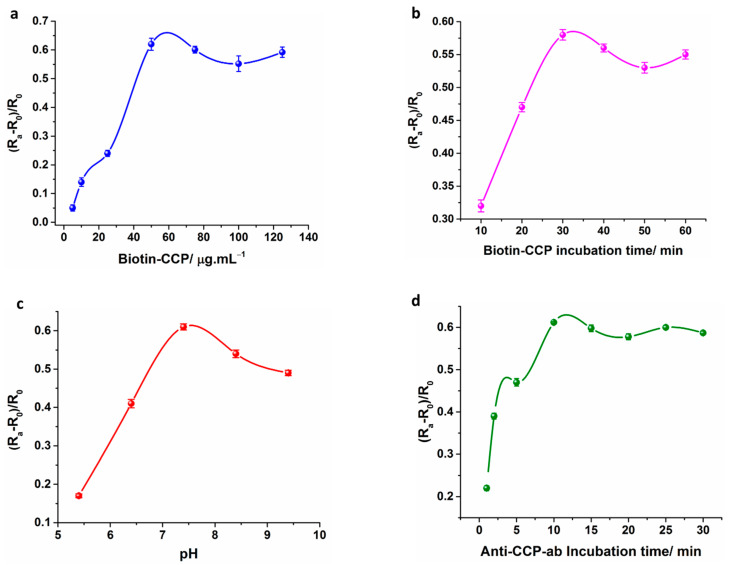
Optimization of the immunosensor for B-CCP concentration (**a**), B-CCP incubation time (**b**) and pH (**c**) and Anti-CCP-ab incubation time (**d**). The data points represent the average of three independent experimental values (*n* = 3), with the range indicated by standard error bars.

The optimization of the electrochemical response is a critical factor to obtain the best sensor performance in terms of linear range, detection limit and sensitivity. The experimental conditions such as Biotin-CCP concentration (5, 10, 25, 50, 75, 100 and 125 µg mL^−1^), Biotin-CCP incubation time (10, 20, 30, 40, 50 and 60 min) and pH (5.4, 6.4, 7.4, 8.4, 9.4) and immunoreaction incubation time (1, 2, 5, 10, 15, 20, 25 and 30 min) of the sensor were optimized, by maintaining the concentration of Anti-CCP-ab as constant (20 IU mL^−1^). In Figure 3a, the sensor was optimized for B-CCP concentration by adding the various concentrations of B-CCP (5 µg mL^−1^ → 125 µg mL^−1^) for sensor fabrication. The impedance change (ΔR_ct_) of the developed sensors increased as the B-CCP loading on the electrode surface is increased from 5.0 to 50 µg mL^−1^, whereas no significant change in the sensor response was detected over 50 µg mL^−1^, due to electrode saturation effect. Hence, a concentration of 50 µg mL^−1^ B-CCP was selected for the later experiments. The optimum B-CCP incubation time for total immobilization of the synthetic peptide was optimized by incubating the B-CCP attached electrode for different time intervals (10 min → 60 min). The impedance signal linearly increased with increase in the B-CCP incubation time up to 30 min, whereas the electrode response was saturated over 30 min of the B-CCP incubation suggesting a 30 min B-CCP incubation is optimum for the sensor fabrication (Figure 3b). In case of pH value optimization, the sensor response is measured in different pH buffers ranging from (pH 5.4 → pH 9.4). The change in impedance (ΔR_ct_) increases with rise in pH value till 7.4, and decreases with the increase in pH value, suggesting that the optimal pH for immunoreaction is 7.4 (Figure 3c). However, at extreme acidic or alkaline pH conditions the proteins tend to denature and aggregate. The optimization of the immunoreaction incubation time between Biotin-CCP and Anti-CCP-ab was carried out for different time periods (1 → 30 min). The ΔR_ct_ increases with rise in immunoreaction incubation time till 10 min, and the signal is not significantly increased above the 10 min of Anti-CCP-ab incubation, demonstrating that the optimum incubation time for the immunoreaction is 10 min (Figure 3d). In precise the optimized sensor conditions are a pH value of 7.4, 50 µg mL^−1^ of B-CCP concentration, a 30 min B-CCP incubation time and a 10 min Anti-CCP-ab incubation time.

### 3.3. Electrochemical Characterization of the ICE Modified Electrode

#### 3.3.1. Electrochemical Impedance Spectroscopy (EIS)

**Figure 4:**
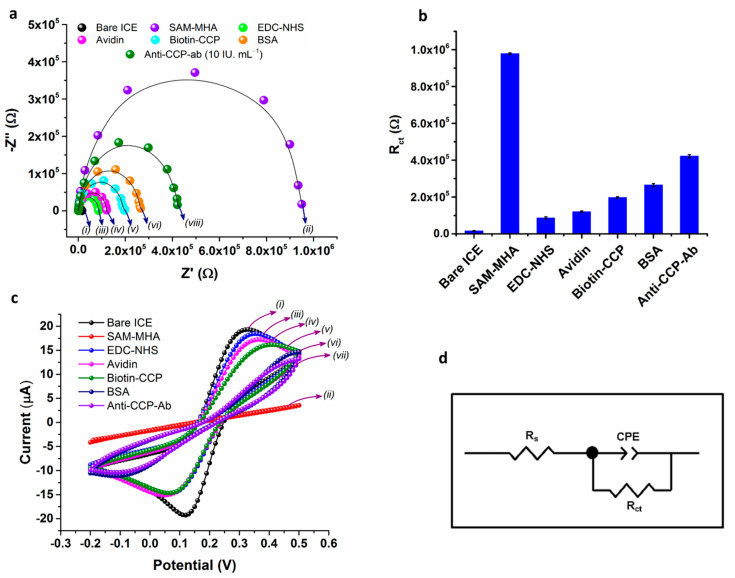
Electrochemical impedance spectra and Cyclic voltammograms of the ICE at major stages of the modification (**a**,**c**). The measurements were carried out in 5 mM [Fe(CN)_6_]^3−^ prepared in 1 × PBS (10 mM), at room temperature (RT) (24 °C). The bar graph for the changes in Rct of the bare and modified ICE electrode at different steps of electrode modification (**b**). The simplified randles-sevcik equivalent circuit model used for the fitting of the EIS spectra measured in the present study (**d**). The data points represent the average of three independent experimental values (*n* = 3), with the range indicated by standard error bars.

Impedance spectroscopy was employed to probe the electrode interfacial property on the ICE surface during the major stages of the electrode assembly. Figure 4a displays the measured impedance spectra represented as Nyquist plots (−*Z*″ vs. *Z*′) acquired on ICE electrodes during step-wise modification of the electrode in a background solution of 5 mM [Fe(CN)_6_]^3−^/^4−^ (1:1) in 1 × PBS. The electrode interfacial property (charge-transfer resistance: *R*_ct_) was obtained by fitting the measured EIS spectra to an equivalent-circuit model (ZView; Scribner Associates Inc., Southern Pines, USA) as shown in Figure 4d. The EIS spectra of the bare ICE surface (Figure 4a, curve *i*) displays a small semicircle with R_ct_ of 15640 Ω, which is characteristic for a bare gold electrode with no diffusional limiting process at lower frequencies, which is an additional benefit of the developed ICE microelectrodes. The diameter of the semicircle was substantially increased with a R_ct_ of 978,900 Ω, due of the surface modification of ICE with mercaptohexanoic acid (Figure 4a, curve *ii*), suggesting that the alkanethiol film created an electrical hindrance for the electrons transfer between the [Fe(CN)_6_]^3−^/^4−^ redox probe and the electrode surface. The semicircle diameter was significantly decreased with a R_ct_ of 86,520 Ω (Figure 4a, curve *iii*), after the EDC-NHS carbodiimide coupling reaction with the terminal –COOH of MHA-SAM, suggesting that formation of succinimide ester on the SAM modified electrode surface facilitating the fast electron transfer between the electrode and the interface. The semicircle diameter is slightly increased (R_ct_: 120,400 Ω) with the linking of avidin on the SAM surface, due to the development of electron-blocking layer on the ICE surface by successful immobilization of avidin (Figure 4a, curve *iv*). The ICE/MHA/EDC-NHS/B-CCP impedance spectrum shows an increase in diameter of semicircle with a R_ct_ of 197,400 Ω (Figure 4a, curve *v*) due to the formation of electron transfer obstruction layer on the electrode surface by the immobilization of B-CCP synthetic peptide, which is a highly non-conductive biological material. The rinsing of the electrode with 1 × PBS buffer containing 0.05% Tween-20 and 0.5% BSA for 2 min has again increased (R_ct_: 265,500 Ω) the diameter of the semicircle (Figure 4a, curve *vi*), due to the binding of BSA on ICE surface to prevent non-specific binding of the Anti-CCP-ab onto the ICE surface. Figure 4a curve *vii* shows a great increase in diameter of the semicircle (R_ct_: 421,700 Ω) when incubated with the Anti-CCP-ab for 10 min, suggesting the binding of analyte with the target and results in decrease in electron transfer rate by formation of ferrocyanide transport obstruction layer between the electrode surface and the electrolyte. The measured impedance spectra are consistent with the CV results (Figure 4c) demonstrating that the B-CCP has bound to the covalently attached avidin and the ICE/MHA/EDC-NHS/B-CCP/BSA immunosensor is successfully developed. Table 1 summary of the extrapolated fitting results of the measured EIS spectra for the circuit elements shown in Figure 4d.

#### 3.3.2. Cyclic Voltammetry (CV)

CV of the bare and modified ICEs were recorded in a solution of 5 mM [Fe(CN)_6_]^3−^/^4−^ (1:1) in 1 × PBS to measure the variations in electrode behavior introduced at major stages of electrode modification (Figure 3c). The CV of the bare ICE show a clear electrochemical response for [Fe(CN)_6_]^4−/3−^ with an oxidation and reduction peak centered at +0.33 V and +0.12 V respectively, as expected (Figure 3c, curve *i*). The electrochemical response was drastically changed and the anodic and the cathodic peak currents were intensely altered, after functionalization of the ICE with MHA-SAM when compared to bare ICE (Figure 3c, curve *ii*). The effect is due to the slow electron transfer kinetics induced by the formation of a high degree of the insulation layer developed by the SAM functionalization. The CV of the ICE/MHA/EDC-NHS shows regeneration of the redox peak for the [Fe(CN)_6_]^3−^/^4−^ (Figure 3c, curve *iii*). The effect is due to the formation of succinimide ester on the electrode surface which results in fast-electron transfer kinetics between the electrode and electrolyte (Figure 3c, curve *iv*). The CV of ICE/MHA/EDC-NHS/B-CCP electrode, ICE/MHA/EDC-NHS/B-CCP/BSA electrode and ICE/MHA/EDC-NHS/B-CCP/BSA/Anti-CCP-ab shows a reduced electrochemical response in compared to the ICE/MHA/EDC-NHS electrode, due to the immobilization of biologically active synthetic peptide (B-CCP), BSA and Anti-CCP-ab binding on the corresponding electrodes, which results in very low electron transfer kinetics and a greatly decreased in the ferricyanide response on the electrode surface (Figure 3c, curve *v-vii*).

### 3.4. Electrochemical Response Studies of the Modified ICE Bioelectrode

**Figure 5:**
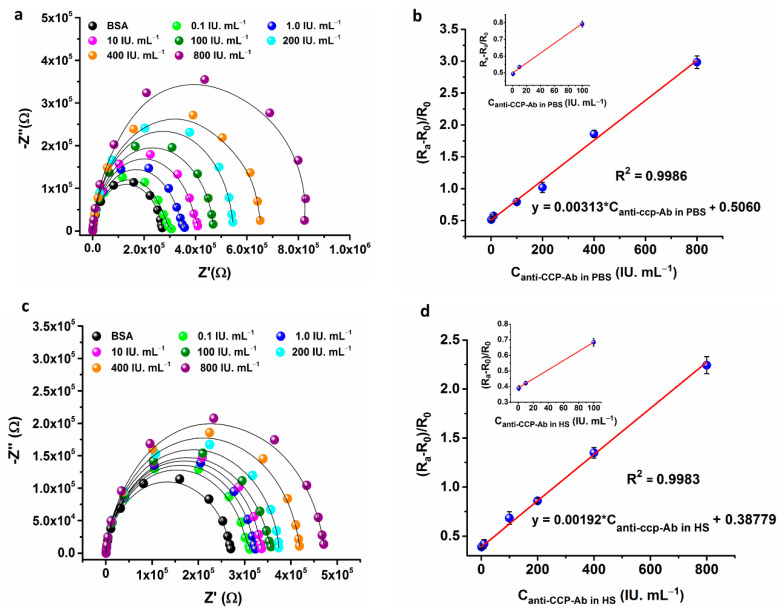
EIS response study of the ICE/MHA/EDC-NHS/B-CCP/BSA bioelectrode with various concentrations of Anti-CCP-ab in PBS and HS respectively (**a**,**c**). The linear calibration curves for the impedance response in PBS and HS with regression equations (**b**,**d**). The inset shows the data points of the calibration curves at lower concentrations of the Anti-CCP-ab. The data points represent the average of three independent experimental values (*n* = 3), with the range indicated by standard error bars.

The binding of Anti-CCP-ab on the modified ICE surface and change in the electrode interfacial impedance was sensitively detected through impedimetric measurements of the electrodes in a frequency range of 0.1 Hz to 1 MHz. The change is impedance was detected by fitting the measured spectra to an equivalent circuit model (Figure 3d), where the changes in electrode interfacial impedance is calculated in terms of Charge-transfer resistance (R_ct_). The developed Anti-CCP-ab immunosensor was well characterized by measuring the changes in R_ct_, which represents the resistance of the electrode at lower frequencies. The results suggest that the change in R_ct_ of the modified ICE electrode upon anti-CCP-ab addition is a critical factor for the quantitative determination of AAb in PBS and HS. Hence, the R_ct_ was measured for a range of Anti-CCP-ab concentrations (0.1 IU mL^−1^ → 800 IU mL^−1^) on the modified electrode. The sensor response was represented as change in R_ct_ denoted as ΔR_ct_. The ΔR_ct_ was calculated by using the formula ΔR_ct_ = (R_a_ − R_0_)/R_0_; where R_a_ and R_0_ are the R_ct_ of the modified electrode in the presence and absence of the analyte respectively. To assess the electrode response to Anti-CCP-ab addition, the developed ICE/MHA/EDC-NHS/B-CCP/BSA bioelectrode was incubated with different concentrations of Anti-CCP-ab prepared in 1 × PBS for 10 min. The impedance measurements were then carried out in 5 mM [Fe(CN)_6_^−3/–4^] (1:1) redox probe prepared in 1× PBS. As shown in Figure 5a, the R_ct_ values linearly increased with increasing Anti-CCP-ab concentration. The ΔR_ct_ was also proportionate to the linear increase in Anti-CCP-Ab concentrations on the electrode over a range of 1 IU mL^−1^ → 800 IU mL^−1^ (Figure 5b). The regression equation for the sensor response in PBS was ΔR_ct_ = 0.5060 + 0.00313*C_anti-ccp-ab(PBS)_ with a correlation coefficient of 0.9986 (Figure 5b).

Moreover, the calculated limit of detection (LOD) of the sensor was 60 mIU mL^−1^ (S/N = 3). The LOD was calculated by using an expression (3*SE/slope) [44]. where SE is calculated by dividing the standard deviation of the blank, by the square root of the number of blank observations [40] and slope is the sensitivity of the calibration curve. The sensor shows a LOD of 0.6 IU mL^−1^ (PBS). The EIS response studies clearly shows that R_ct_ increased linearly with step wise increase of Anti-CCP-ab concentration on the electrode surface. The effect can be explained by the formation of a kinetic barrier for the transfer of electrons due to the step-wise increase in the concentration of Anti-CCP-ab, which binds to the immobilized B-CCP on the SAM modified ICE surface. The ΔR_ct_ of the modified ICE electrode was adopted as a parameter for the quantification of the sensor signal to detect Anti-CCP-ab via specific immunoreactions on the electrode surface. In this fashion, the developed bio-electrode established a proof-of-concept for the direct and label-free detection of Anti-CCP-ab without further amplification of the electrical signal. The immunosensor displayed a good dynamic ranges and detection limits in comparison to the other Anti-CCP-ab biosensors reported in the past decade (Table 2).

In order to construct a good linear calibration curve and to avoid the matrix effects a dilute human serum solution (1% *v/v*) in 1 × PBS buffer was prepared. HS containing different concentrations of Anti-CCP-ab were loaded on to the modified ICE electrode surface and incubated for 10 min in a humid chamber to react with B-CCP immobilized on the electrode surface. After incubation with HS containing different concentrations of AAb, impedance spectra were measured in 5 mM [Fe(CN)_6_^−3/−4^] electrolyte solution. In comparison to the blank electrode, HS (anti-CCP-ab) added electrode impedance was increased with linear increase in concentration of analyte (Figure 5c). The ΔR_ct_ was also proportionate to the linear increase of Anti-CCP-ab concentration over a range of 0.1 IU mL^−1^ → 800 IU mL^−1^. The regression equation for sensor response in HS was ΔR_ct_ = 0.3877 + 0.00192*C_anti-ccp-ab(HS)_ with a correlation coefficient of 0.9983 (Figure 5d). Moreover, the calculated limit of detection (LOD) of the sensor was 0.82 IU mL^−1^ (S/N = 3). The impedance signal measured with HS samples are dependent on concentration of Anti-CCP-ab. The calibration curve constructed for determination of Anti-CCP-ab in HS is used for analysis of real samples.

### 3.5. Application of the Bioelectrode

#### 3.5.1. Interference Study

**Figure 6:**
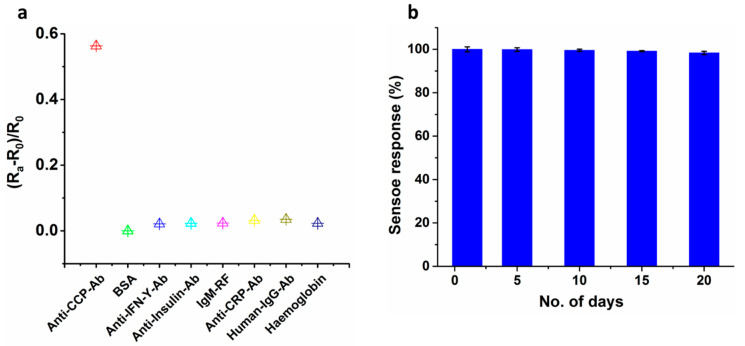
Effect of interferents in the absence (control) and presence of different co-existing interfering agents present in HS (**a**). The storage stability of the ICE/MHA/EDC-NHS/Avidin/B-CCP/BSA bio-electrode carried out for a period of 20 days at a regular interval of 5 days (**b**). The electrodes were stored at 4 °C when not in use. The data points represent the average of three independent experimental values (*n* = 3), with the range indicated by standard error bars.

The interference of the ICE/MHA/EDC-NHS/B-CCP/BSA bioelectrode is studied by measuring the impedance signal in the presence and absence of different proteins co-exist in the HS. The interfering proteins selected for the study are Anti-IFN-γ-ab (100 pg mL^−1^), Anti-insulin-ab (200 pg mL^−1^), IgM-RF (50 IU mL^−1^), Anti-CRP-ab (200 pg mL^−1^), Human-IgG-ab (100 pg mL^−1^), Hemoglobin (15 mg mL^−1^), Anti-CCP-ab (10 IU mL^−1^). The concentration of the interferents selected is two folds greater than the expected concentration levels of the healthy individuals. These interferents prepared in 1 × PBS are added to the ICE/MHA/EDC-NHS/B-CCP/BSA bioelectrode separately and electrode response is obtained in a frequency range of 0.1 Hz–1 MHz. Figure 6a shows that no significant interference was detected in the determination of Anti-CCP-ab in the presence of the selected interring agents. The results established that the high specificity of the immobilized B-CCP synthetic peptide used for the detection of the target analyte and a good selectivity of the electrochemical impedance transducer at the optimized experimental conditions.

#### 3.5.2. Stability Study

The freshly prepared ICE/MHA/EDC-NHS/B-CCP/BSA bioelectrode was stored in 1 × PBS at 4 °C and the impedance signal was measured as a function of time. The stability of the developed ICE/MHA/EDC-NHS/B-CCP/BSA bioelectrode was assessed by measuring the R_ct_ at a regular intervals of 5 days for 20 days. Figure 6b shows that the developed bioelectrode retained ~98.5% of its activity at the end of 20 days.

#### 3.5.3. Reproducibility

Reproducibility is an important factor for the potential application of the immunosensor in clinical diagnostics. Thus, the reproducibility of the fabricated ICE/MHA/EDC-NHS/B-CCP/BSA bioelectrode was verified by sensing Anti-CCP-ab (10 IU mL^−1^) on three independently prepared ICE/MHA/EDC-NHS/B-CCP/BSA bio-electrodes. The RSD calculated for the three measurements was 1.52%, suggesting that the fabricated immunosensor shows an acceptable reproducibility. The reproducibility for the three different ICE/MHA/EDC-NHS/B-CCP/BSA bioelectrode (R_0_) was also verified in 1 × PBS, and the results demonstrated a RSD of 1.02%. A very good reproducibility and stability is endorsed due to a robust Avidin-Biotin interaction system and covalent immobilization of avidin molecules on the gold surface, which created a bio-compatible substrate and prevents the removal of bio-receptors from the modified ICE surface. moreover, the presented impedimetric interdigitated microelectrode array sensor shows several advantages compared to the other reported methods for CCP detection. The developed assay (a) is label-free, (b) mass production of ICE at low cost, (c) no Warburg diffusion at lower frequencies, (d) requirement of low sample volumes (10 µL), (e) low-cost sample preparation, (f) a low assay time and (e) direct signal readout in the form of an electrical signal.

### 3.6. Real-Sample Analysis

To validate the practical application of the developed ICE/MHA/EDC-NHS/B-CCP/BSA bioelectrode, Anti-CCP-ab was quantified in spiked HS samples. The analysis was carried out by spiking five different concentrations of Anti-CCP-ab (1.0, 10, 20, 50 and 500 IU mL^−1^) into 1% *v*/*v* PBS diluted HS samples. The HS samples spiked with bio-analyte were loaded onto the developed ICE/MHA/EDC-NHS/B-CCP/BSA bio-electrode. Three different measurements for each concentration was obtained and the average of three measurements was calculated and the recovery rates with the %RSD was tabulated. In Table 3, the measured Anti-CCP-ab concentration in spiked human serum samples using the developed immunosensor was found to be 0.98, 9.95, 19.98, 50.59 and 499.02 IU mL^−1^. The calculated recoveries are 98.0% (*n* = 3), 99.9% (*n* = 3), 99.5% (*n* = 3), 101.18% (*n* = 3) and 99.80 (*n* = 3), respectively. The results suggest that the biosensor shows a good recovery rate for the five samples with no significant variation between the spiked and the found Anti-CCP-ab concentrations. Thus, the ICE/MHA/EDC-NHS/B-CCP/BSA bioelectrode fabricated can be potentially used for the accurate quantification of the Anti-CCP-ab’s in the real samples.

## 4. Conclusions

Miniaturized electrochemical impedance immunosensor based on Interdigitated microelectrode array for selective detection of Anti-CCP-ab was developed through a simple Biotin-Avidin recognition system. The avidin is attached on the ICE surface by modification of electrode with MHA-SAM and the functionalization of the ICE with SAM is characterized through AFM, EDX, CV, XPS, EIS. SAM provided a bio-compatible environment to attach and preserve the bio-activity of the B-CCP. The Avidin-Biotin recognition system provided significant stability to the CCP bound onto the electrode surface. The sensor is successfully validated as an impedimetric biosensor for the quantification of Anti-CCP-Ab in PBS and HS. The biosensor showed a linear range of 1 IU mL^−1^ → 800 IU mL^−1^ and a detection limit of 0.60 IU mL^−1^ (PBS). Additionally, the microscale design of the sensor (3.5 × 14 mm) enabled the ICE to easily assemble with a portable-potentiostat making the bio-electrode suitable for POC testing. The fabricated bioelectrode is economical and feasible for mass production and the detection has not required any fluorophores. It sensor can applied for detection of arthritis AAb (Anti-CCP-ab) in individuals at early stages of disease development.

## Figures and Tables

**Table 1:** EIS parameters of the bare and modified ICEs extrapolated by fitting the measured spectra to the equivalent circuit model shown in Figure 3d.

Electrode	R_S_ [Ω]	CPE		R_ct_ [Ω]	Chi Square
		T [Ω^−1^. s^p^]	P		
Bare ICE	575.1	2.450 × 10^−7^	0.85416	15,640	0.0545
MHA	550.6	1.594 × 10^−7^	0.90534	978,900	0.0330
EDC-NHS	552.8	1.266 × 10^−7^	0.92107	86,520	0.0343
Avidin	486.0	9.487 × 10^−8^	0.93138	120,400	0.0291
B-CCP	562.3	1.361 × 10^−7^	0.92019	197,400	0.0300
BSA	568.6	1.577 × 10^−7^	0.92658	265,500	0.0291
Anti-CCP-ab	527.1	9.478 × 10^−8^	0.93961	421,700	0.0276

**Table 2:** Comparison of the present Anti-CCP-ab immunosensor analytical figures-of-the-merit with other reported works.

Detection Assay	Linear Range (IU mL^−1^)	LOD (IU mL^−1^)	Sample Type	Reference
Electrochemical Amperometric	10–1000	2.5	PBS	[19]
Fluorescence immunoassay (BioPlex^TM^ 2200)	3–300.0	0.2	HS	[14]
ELISA (ImmunLisa^TM^ CCP)	25–3200.0	1.6	HS	[15]
Electrochemiluminescence	0.041–6.26	0.008	PBS	[45]
EIS	1–800	0.60	PBS	This work
EIS	1–800	0.82	HS	This work

**Table 3:** Recovery study of the ICE/MHA/EDC-NHS/B-CCP/BSA biosensor using the HS spiked with different concentrations of Anti-CCP-ab.

Test Sample	Conc. Of Anti-CCP-ab in Diluted HS (IU mL^−1^)	Spiked (IU mL^−1^)	Found (IU mL^−1^)	Recovery (%)	RSD (%)
A	0	1	0.98	98.0	1.09
B	0	20	19.98	99.9	1.15
C	0	10	9.95	99.5	1.27
D	0	50	50.59	101.18	1.92
E	0	500	499.02	99.80	1.40

## Data Availability

No new data were created or analyzed in this study. Data sharing is not applicable to this article.

## Supplementary Materials

The following are available online at https://www.mdpi.com/1424-8220/21/1/124/s1, Figure S1. Energy dispersive x-ray spectroscopy of the bare ICE and the 6-Mercaptohexanoic acid modified ICE surface; Figure S2. X-ray photoelectron spectroscopy survey spectra of the 6-mrcaptohexanoic acid functionalized on the electrode surface.
