# Supplementary material for: Electrochemical Immunosensor for the Early Detection of Rheumatoid Arthritis Biomarker: Anti-Cyclic Citrullinated Peptide Antibody in Human Serum Based on Avidin-Biotin System
Source: Sensors (Basel). 2020 Dec 28;21(1):124. doi: 10.3390/s21010124 (PMC7795521; doi:10.3390/s21010124)
Supplement: Supplementary file 1 [file sensors-21-00124-s001.pdf]

sensors

MDPI

Article

Electrochemical Immunosensor for the Early Detection of Rheumatoid Arthritis Biomarker: Anti-Cyclic Citrullinated Peptide Antibody in Human Serum Based on Avidin-Biotin System

Somasekhar R. Chinnadayyala [1] and Sungbo Cho [1,2,*]

[1] Department of Electronic Engineering, Gachon University, 1342 Seongnamdaero, Seongnam-si, Gyeonggi-do 13120, Korea; ssreddy@gachon.ac.kr
[2] Department of Health Science and Technology, GAIHST, Gachon University, Incheon 21999, Korea
* Correspondence: sbcho@gachon.ac.kr; Tel.: +82-(31)-750-5321

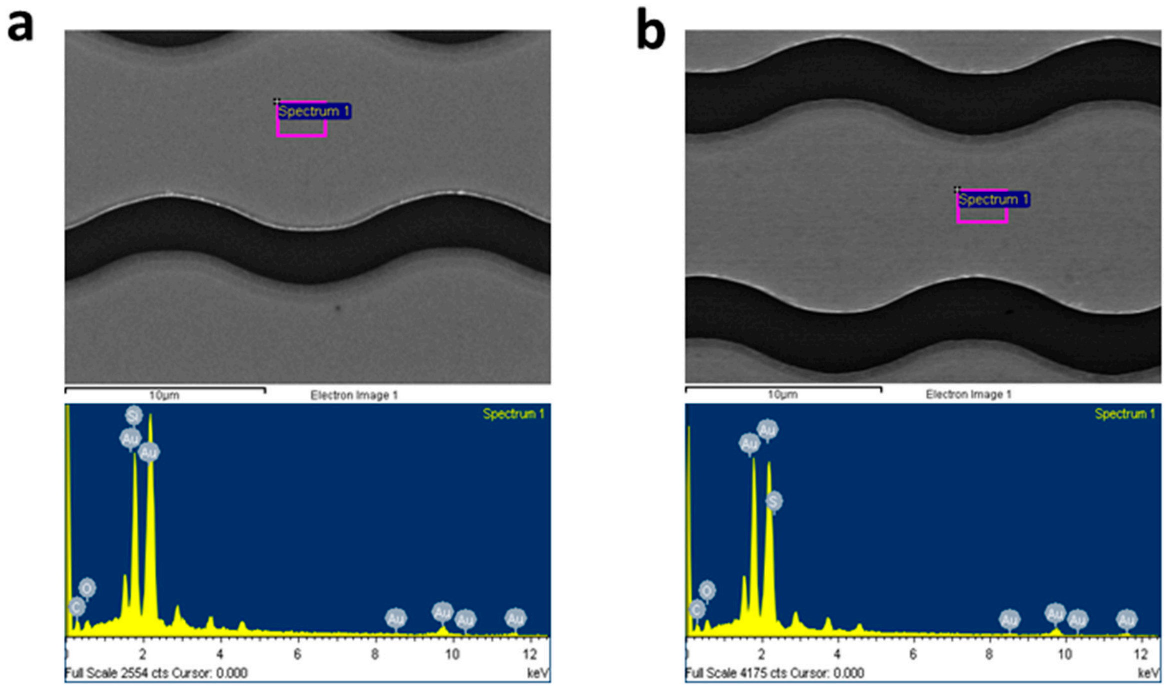

(a)

Element	Atomic%
C K	7.59
O K	14.35
Si K	5.39
Au M	72.67
Totals	100

(b)

Element	Atomic%
C K	39.36
O K	32.87
S K	1.60
Au M	26.17
Totals	100

Figure S1. Energy dispersive x-ray spectroscopy of the bare ICE (**a**) and the 6-Mercaptohexanoic acid modified ICE surface (**b**).

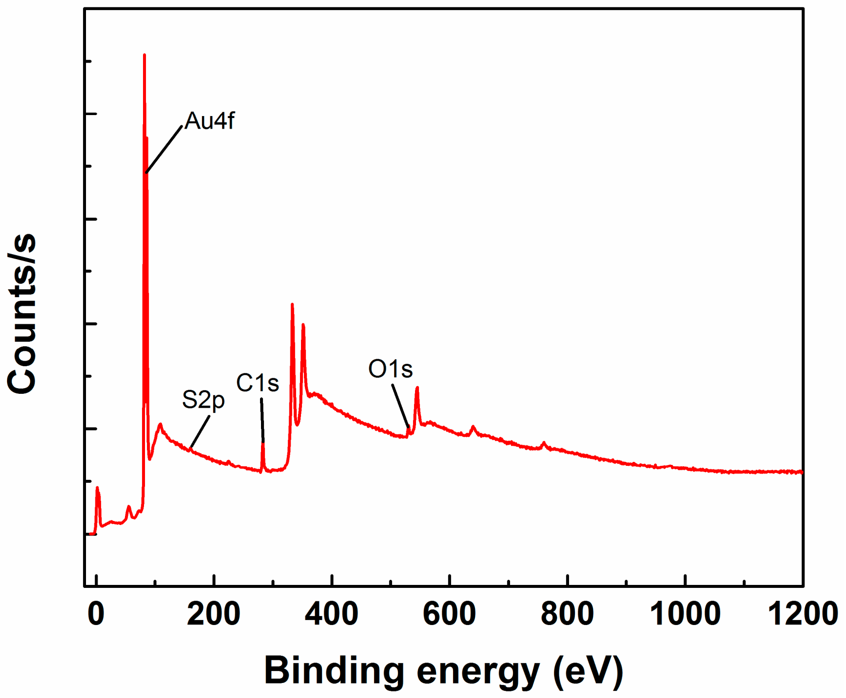

Figure S2. X-ray photoelectron spectroscopy survey spectra of the 6-mrcaptohexanoic acid functionalized on the electrode surface.
